# Identifying ataxia‐telangiectasia in cancer patients: Novel insights from an interesting case and review of literature

**DOI:** 10.1002/ccr3.3543

**Published:** 2020-12-30

**Authors:** Jinyi Cao, Ryan Ying Cong Tan, Shao‐Tzu Li, Eliza Courtney, Ronald Chin Hong Goh, Bingwen Eugene Fan, Kiattisa Sommat, Ravichandran Nadarajah, Joanne Ngeow

**Affiliations:** ^1^ Division of Medical Oncology National Cancer Centre Singapore Singapore Singapore; ^2^ Department of Obstetrics & Gynaecology Singapore General Hospital Singapore Singapore; ^3^ Oncology Academic Clinical Program Duke‐NUS Graduate Medical School Singapore Singapore; ^4^ Cancer Genetics Service Division of Medical Oncology National Cancer Centre Singapore Singapore Singapore; ^5^ Department of Anatomical Pathology Singapore General Hospital Singapore Singapore; ^6^ Department of Haematology Tan Tock Seng Hospital Singapore Singapore; ^7^ Department of Laboratory Medicine Khoo Teck Puat Hospital Singapore Singapore; ^8^ Yong Loo Lin School of Medicine Singapore Singapore; ^9^ Lee Kong Chian School of Medicine Singapore Singapore; ^10^ Division of Radiation Oncology National Cancer Centre Singapore Singapore Singapore

**Keywords:** ataxia‐telangiectasia, cancer genetics, cancer management

## Abstract

Timely genetic testing leading to early diagnosis of A‐T is crucial due to its plethora of implications on clinical management, particularly in those who develop malignancies. Thus, clinicians have to be astute in identifying diagnostic clues of A‐T.

## Graphical abstract

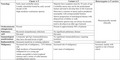

## 1 INTRODUCTION

Ataxia‐telangiectasia (A‐T), a rare hereditary cancer syndrome, can present with a myriad of clinical manifestations. Here, we described a case whose diagnosis of A‐T was missed till a second malignancy was confirmed. We aim to highlight diagnostic clues of A‐T and discussed important considerations in management of malignancies in A‐T.

Ataxia‐telangiectasia (A‐T) (OMIM #208900) is a rare autosomal recessive disorder resulting from biallelic pathogenic variants in the Ataxia‐Telangiectasia mutated (*ATM)* gene (OMIM *607585). Classically, it is characterized by progressive cerebellar ataxia, cutaneous telangiectasia, immunodeficiency, cancer susceptibility and radiation sensitivity.^1^, ^2^, ^3^ However, variant A‐T may have a myriad of presentations. The *ATM* gene encodes a serine/threonine protein kinase which plays a crucial role in the repair of DNA double‐stranded breaks^1^, ^2^, ^3^, ^4^, ^5^ and when impaired leads to carcinogenesis. Studies estimate lifetime cancer risks of 25%‐40%^4^, ^5^, ^6^ of both solid and hematological malignancies.^2^, ^4^, ^7^, ^8^, ^9^, ^10^ Here, we report a patient with sensorimotor polyneuropathy, metachronous T‐cell prolymphocytic leukemia (T‐PLL), and cervical carcinosarcoma who was eventually diagnosed with A‐T to highlight clinical pearls and important management considerations for clinicians.

## 2 CASE REPORT

**FIGURE 1:**
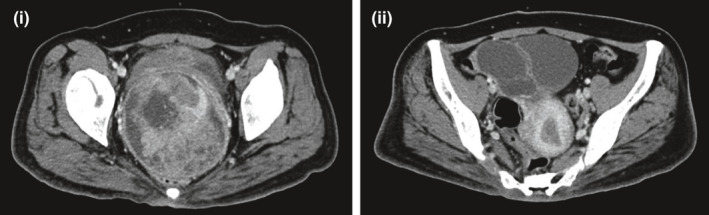
Computed tomography abdomen pelvis scan showing (i) Cervical tumor (ii) Ovarian metastases

**FIGURE 2:**
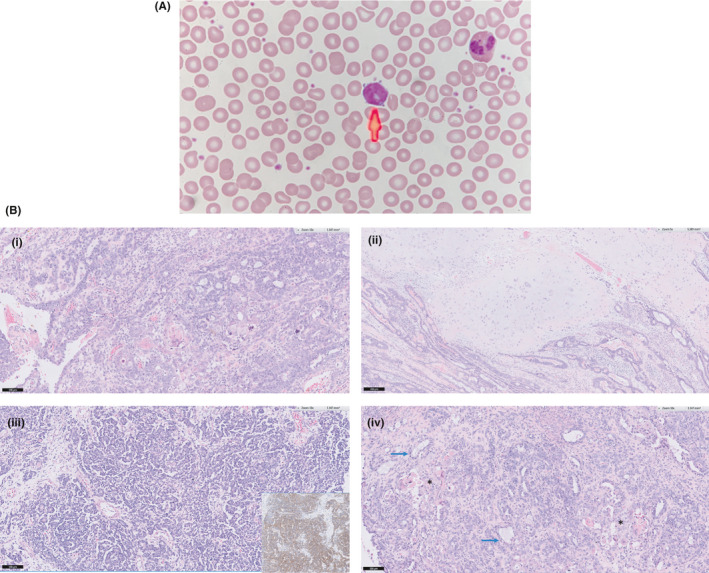
A, Mature‐looking T‐PLL lymphocyte with cytoplasmic blebs in peripheral blood film. B, Histology slides showing (i) Cervical adenosquamous carcinoma with complex glandular proliferation, papillae, and scattered keratinizing squamous whorls, (ii) Focal malignant cartilaginous nodules associated with cervical adenocarcinoma, (iii) Anastomosing cords of cells punctuated by small tubules, demonstrating cytoplasmic reactivity for synaptophysin on immunostain, and (iv) Ovarian metastasis with similar looking adenosquamous carcinoma with foci of keratinization (*) and some cells containing cytoplasmic mucin (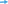)

A 34‐year‐old Chinese woman presented with urinary incontinence, intermittent abdominal discomfort, and menorrhagia. On physical examination, a necrotic bleeding vaginal mass was noted. Computed tomography (CT) of the abdomen and pelvis revealed a cervical lesion and right ovarian lesion measuring 8 × 7 cm and 9 × 6 cm, respectively Figure 1. Medical history was significant for possible cerebral palsy that was recently revised to possible Charcot‐Marie‐Tooth disease when she presented with progressively worsening weakness, with nerve conduction study and electromyography showing diffused sensorimotor axonal polyneuropathy. She was diagnosed with CD4/CD8 double‐positive T‐PLL Figure 2A with complex cytogenetics at age 31 after an incidental finding of leucocytosis. Bone marrow cytogenetics then showed an abnormal mosaic female chromosome analysis with a normal cell line and one with numerical and structural abnormalities. However, there were no deletions or missense variants involving the *ATM* locus 11q23, which is present in up to 65% of all cases of T‐PLL.^11^, ^12^, ^13^ She was placed on expectant management given absence of cytopenia nor rapidly increasing lymphocytosis, B symptoms, lymphadenopathy, or end organ involvement, as per T‐PLL International Study Group (TPLL‐ISG) guidelines.^14^ Moreover, given her comorbidities and functional status, she was a poor candidate for most cytotoxic treatments targeting T‐PLL or bone marrow transplant. Other comorbidities included type 2 diabetes mellitus, multiple ophthalmological issues and persistently raised alpha‐fetoprotein (AFP) with mild transaminitis since age 29 for which investigations were unyielding.

Tumor markers were normal apart from baseline elevated AFP: CEA 1.5 ug/L, CA 125 23.6 u/ml, Beta‐hCG < 0.6U/l, AFP 153 ug/L. Cervical biopsy showed squamous cell carcinoma (SCC) while right ovarian biopsy showed adenocarcinoma with focal mucinous differentiation. Our multidisciplinary consensus was that of at least FIGO stage IIB cervical SCC with a synchronous primary ovarian mucinous adenocarcinoma. Initial recommendation was made for definitive treatment with neoadjuvant chemotherapy followed by chemo‐radiotherapy for her cervical SCC and sequential debulking surgery for her ovarian adenocarcinoma. She received 2 cycles of paclitaxel (175 mg/m^2^) and carboplatin (AUC 5) at 3 weekly intervals but did not achieve adequate control of her symptoms of pain and per vagina bleeding. After restaging scans showed local progression of the ovarian mass, she underwent palliative open radical hysterectomy, bilateral salpingo‐oophorectomy, and bilateral pelvic lymphadenectomy. Histology revealed cervical carcinosarcoma with heterologous (cartilaginous) differentiation, predominantly comprised of adenosquamous carcinoma with focal neuroendocrine differentiation, admixed with a minor sarcomatous component. There was bilateral parametrium, upper vagina, lower uterine segment, and pelvic lymph node involvement. Histology of the right ovarian lesion revealed adenosquamous carcinoma, favoring metastasis from the cervical tumor as both had similar histology and immunoprofile Figure 2B.

**FIGURE 3:**
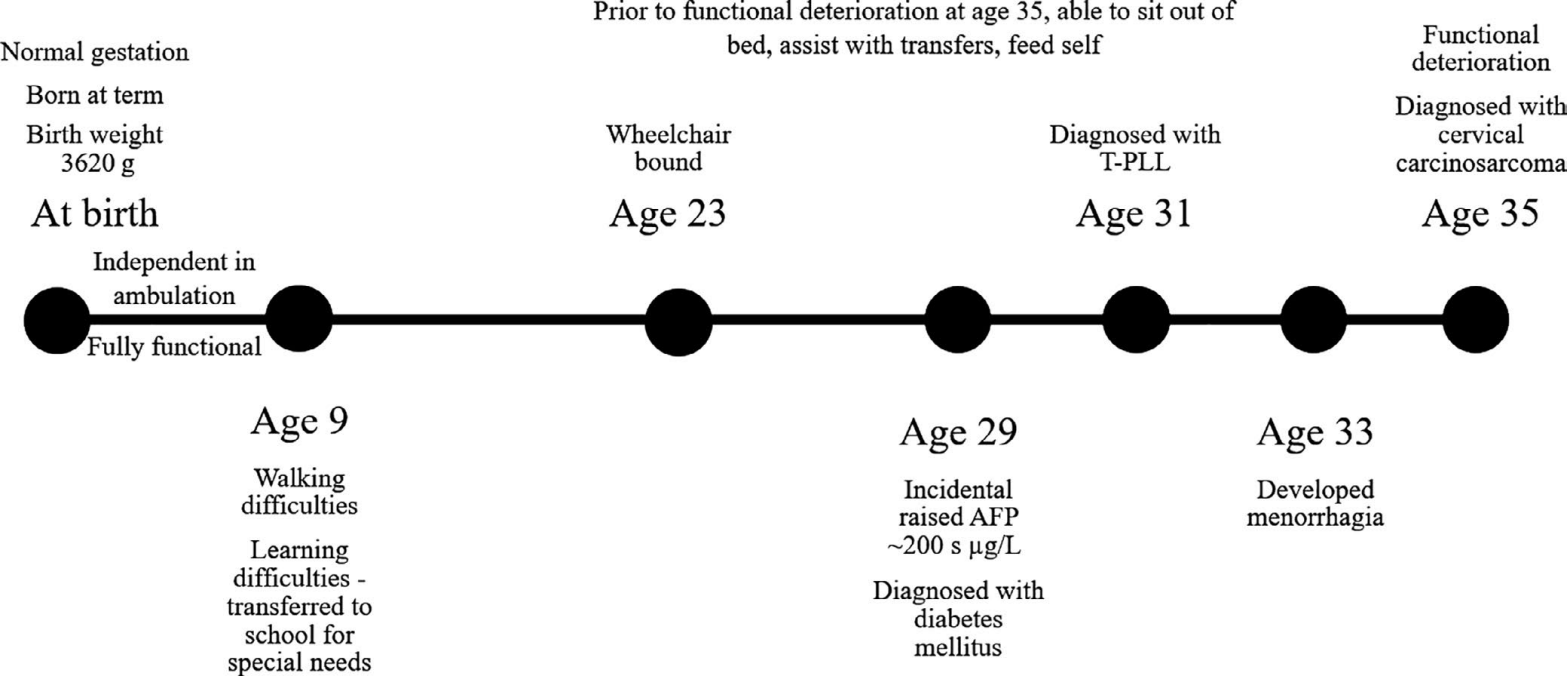
Timeline of events

**FIGURE 4:**
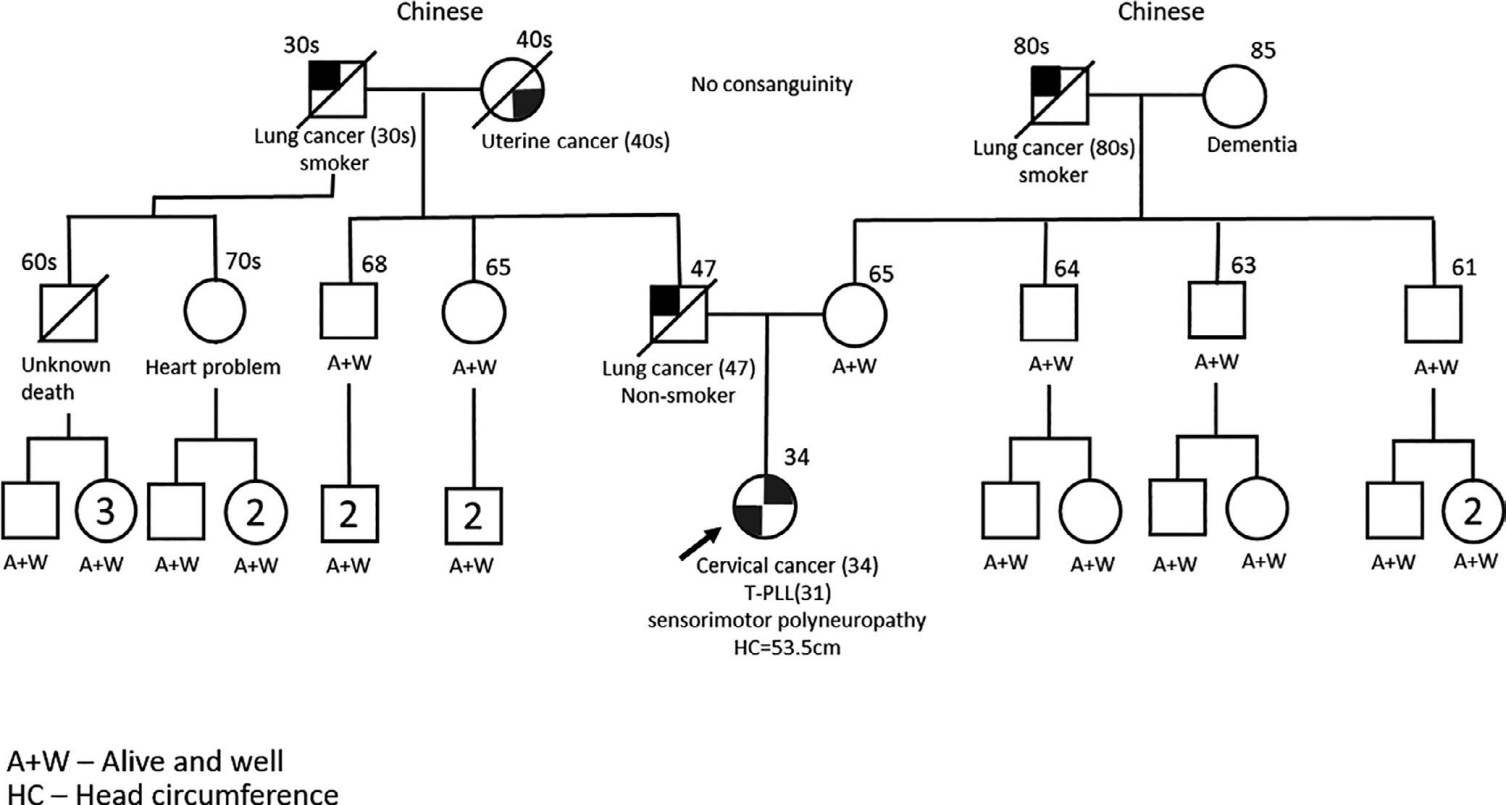
Pedigree

In view of multiple primary cancers at a young age and uncertain underlying neurological condition, she was referred for genetic assessment upon her cervical cancer diagnosis. Born at term, independently ambulant and fully functional initially, she subsequently had difficulty walking and learning around age 9 and became wheelchair bound since age 23 Figure 3. Interview with patient's caregiver revealed that she was thought to have cerebral palsy, and her initial clinicians did not consider a diagnosis of A‐T. Unfortunately, specific details regarding her neurological deterioration and the workup then were not available as she was seen in a different institution. There was no family history of consanguinity nor developmental issues. Her father, a nonsmoker, died from lung cancer at age 47. Her paternal grandfather, a smoker, also died from lung cancer in his 30s while her paternal grandmother died from uterine cancer in her 30‐40s Figure 4. Saliva collected for germline clinical multi‐gene panel testing using next‐generation sequencing revealed two pathogenic variants in *ATM* (NM_000051.3): c. 2304_2305insTT (p.Glu769Leufs*9) and c. 9023G > A (p.Arg3008His). Cytogenetic testing performed on patient's blood revealed an increase in both spontaneous and Gamma‐Ray induced chromosome breakage, confirming the diagnosis of ataxia‐telangiectasia. Clinical examination did not reveal any cutaneous telangiectasia, although a broad face with coarse eyebrows and a few café‐au‐lait spots were noted. Subsequent testing revealed low IgG and IgA levels with gross pan‐cerebellar atrophy on magnetic resonance imaging of the brain in keeping with A‐T.

In view of her A‐T diagnosis, it was recommended to avoid radiation therapy in subsequent treatment. A postoperative CT revealed a new right lung nodule and a hepatic lesion likely representing metastases. Her diagnosis was revised to FIGO stage IVB cervical carcinosarcoma and she received a further 2 cycles of palliative chemotherapy with etoposide (300 mg/m^2^) and cisplatin (100 mg/m^2^), with a 50% dose reduction in view of a diagnosis of A‐T. Her disease progressed 3 months later, and she was placed on best supportive care prior to her demise shortly after.

## 3 DISCUSSION

This case highlights the potential for missed or delayed A‐T diagnoses, especially in cases of variant A‐T, and provides an impetus for clinicians to be aware of suggestive signs to facilitate earlier diagnosis. The first clue of our patient's A‐T diagnosis was the regressive loss of developmental milestones from age 9. Second, the early‐onset T‐PLL concurs with A‐T patients having a predisposition to T‐cell as opposed to B‐cell tumors^12^ and at a significantly younger age of 20‐30s^4^, ^10^, ^12^, ^15^, ^16^ compared to a median age of 69 in patients without A‐T.^10^ Third, multiple primary cancers in patients with A‐T are not uncommon, with incidence ranging from 4% to 15%.^8^, ^17^, ^18^ Of note, solid tumors mainly present in adulthood, with majority being breast, liver, gastric, thyroid, and esophageal carcinomas Appendix 1.^2^, ^4^, ^7^ Finally, diabetes mellitus and particularly the raised AFP of unknown cause were also consistent with A‐T.^19^, ^20^, ^21^

A range of phenotypes have been described in individuals with A‐T Table 1. Patients with variant A‐T have residual ATM kinase activity and thus a milder clinical course than classic A‐T.^22^, ^23^ Variant A‐T may present with extrapyramidal signs instead of cerebellar ataxia, milder neurological symptoms, and no lung disease or immunodeficiency. Although residual ATM kinase activity is protective against childhood tumors, variant A‐T are still at increased risk of developing cancers^22^, ^23^ especially solid malignancies given their longer lifespan compared to classic A‐T whose average life expectancy is approximately 25 years^24^ ^,^ emphasizing the importance of timely genetic testing in this group who may present atypically. Although ATM kinase activity was not tested, based on clinical presentation, our patient is likely to have variant A‐T. Furthermore, residual ATM kinase activity has been demonstrated in another patient with the c. 9023G > A (p.Arg3008His) variant.^23^ In comparison, A‐T heterozygotes often have a normal clinical phenotype. Although epidemiological studies report increased incidence of malignancies in blood relatives of A‐T patients^25^, ^26^, ^27^, ^28^, ^29^ only the risk of breast cancer has been consistently shown to be raised, with lifetime risk of approximately 38%.^29^ Female relatives who are A‐T heterozygotes should thus be offered surveillance with yearly mammography starting from age 40.^30^

**TABLE 1:** Differences in features between classic A‐T, variant A‐T and ATM heterozygotes^22^, ^23^, ^29^, ^53^

	Classic A‐T	Variant A‐T	Heterozygotes A‐T carriers
Neurology	Early‐onset cerebellar ataxia	Majority have symptom onset by 10 y of age	Phenotypically normal clinically
	Usually, wheelchair bound by early second decade of life	Cerebellar ataxia may not be the predominant feature and tend to develop later in life if present	
	High incidence oculomotor apraxia	Most have a mixture of ataxia and/or peripheral neuropathy with extrapyramidal features	
		Slower progression of neurological disease with delayed loss of ability to walk	
		Oculomotor apraxia may not always be present, tend to develop at an older age if present	
Oculocutaneous telangiectasia	Present	Present in approximately 60% of patients	
Pulmonary	Recurrent sinopulmonary infections	No significant pulmonary disease	
AFP	Elevated	Elevated	
Immunological manifestations	Commonly IgG/IgA immunodeficiency	No significant immunodeficiency requiring treatment	
	May have elevated levels of IgM		
Radiosensitivity	Increased sensitivity to ionizing radiation	Variable	Controversial
Malignancy	Increased risk of malignancy, ~25% lifetime risk	Increased risk of malignancy	Increased, mainly with regards to risk of breast cancer
	High incidence of hematological malignancies at a young age	Later onset of malignancy	
	Adults susceptible to both lymphoid tumors and a variety of solid tumors including breast cancers		

To our knowledge, this is the first clinical report of an association between A‐T and cervical cancer though it has been reported in relatives of A‐T patients who are obligate heterozygous carriers of *ATM* variants.^9^, ^27^, ^28^, ^31^ The association between somatic alterations in *ATM* and risk of cervical cancer have also been reported.^32^, ^33^ Despite our patient's strong family history of young lung cancers, this has not been prominently reported in clinical literature on A‐T. Interestingly, up to 40% of lung adenocarcinomas have been reported to lack ATM protein expression due to somatic alterations.^34^ *ATM* rs189037, rs664677, and rs664143 gene polymorphisms have also been reported as risk factors for lung cancer.^35^ These *ATM* variants deserve further study with regards to their association with lung cancer, particularly in Asians where there is a higher incidence of adenocarcinomas in nonsmokers.

While radiation‐induced toxicities including death and secondary malignancies^5^, ^36^ are well established in A‐T, evidence is lacking for chemotherapy. Certain chemotherapeutic agents have been shown to have increased toxicities Appendix 2, whereas agents such as prednisone, 6‐mercaptopurine, asparaginase, and daunorubicin have been shown to be tolerable at normal doses.^37^ There are currently no consensus guidelines with regard to dosing of chemotherapy in A‐T. Various approaches tried in multiple hematological and solid cancers are summarized in Table 2. Inferences that can be drawn are limited by the heterogeneity of primary malignancies reported over an extended time course whereby the standard dose/regime may have evolved.^37^, ^38^, ^39^, ^40^ In general, the most common strategy employed across studies is a 50% dose reduction of the standard regime. Some gradually up titrated the dose as tolerated while taking care to limit doses of certain agents, such as methotrexate and cyclophosphamide. Durable complete remissions have been successfully achieved with modified dose chemotherapy regimens.^41^, ^42^, ^43^, ^44^, ^45^, ^46^, ^47^, ^48^, ^49^ The largest of these studies by Schoenaker et al^39^ demonstrated no significant difference in remission rates for patients with T‐cell acute lymphoblastic leukemia receiving modified dose chemotherapy. Studies to better describe safety and efficacy of chemotherapeutic regimes in A‐T patients are needed. Ultimately, the decision regarding treatment regime and dosage should be a discussion among all managing healthcare professionals, patient and their family, and individualized based on patient's underlying comorbidities, functional status, and treatment goals.

**TABLE 2:** Summary of dosages, toxicities and efficacy of chemotherapy in A‐T patients with cancer^37^, ^38^, ^39^, ^40^, ^41^, ^42^, ^43^, ^44^, ^45^, ^46^, ^47^, ^48^, ^49^, ^54^, ^55^, ^56^, ^57^, ^58^, ^59^

Tumor type	Tumor subtypes	Case report/Series	No. of cases on SD chemo	No. of cases on MD chemo	Stage	*Chemotherapy	% Dose reduction	**Toxicities of note**	**Response rates**	**Overall survival (OS)**
Non‐Hodgkin Lymphoma (NHL)	Burkitt's	Sandoval & Swift	7	1	I, II, IV	COP, CHOP, CP, COMV	Ranging from 33% to 75% of SD	^a^7 of 14 (50%) exposed to CPM ≥ 1200 mg/m^2^ had hemorrhagic cystitis ^a^All 3 on bleomycin (both SD and RD) had pulmonary disease which was fatal in 2	Burkitt's: CR in 5 of 7 on SD, 1 on RD did not achieve remission ^a^All study patients: CR in 1 of 11 on RD vs 16 of 21 on SD; *P* = .001	^a^Mean survival of SD vs RD: 12 (1‐162 + mo) vs 5 (0.5‐28 mo); *P* = .03 ^a^Median survival with CR vs no CR on SD: 32.5 (1‐162 + mo) vs 5 (1‐22 mo); *P* = .01
		Ben Arush et al	1	0		COMP		Died of severe pneumonia 1 mo later		1 mo
		Bienemann et al	0	2	III, IV	B‐NHL‐BFM 04	50% SD Increased to 75% after 2 cycles for 1 pt VP16 omitted for cycle 1, MTX at 0.5 g/m^2^ for 1 pt		At least 1 with CCR	
		Upadhyaya et al	0	1	I	CPM, VCR, DOX, Pred	VBL instead of VCR on D6	Neutropenia, mucositis—2nd cycle DOX reduced to 75% of SD		EFS at least 6 y
	Large cell	Sandoval & Swift	7	4	I‐IV	CHOP, COP, OH, OP, CHVP, HOP, CO, MTX	Ranging from 33% to 75% of SD	See above	CR in 5 of 7 on SD, 4 of 4 on RD did not achieve remission	See above
	Immunoblastic large cell	Ben Arush et al	1	0		COMP		Died 1 mo later of Acinetobacter sepsis		1 mo
	Lymphoblastic	Sandoval & Swift	2	1	III, IV	CHOP, COP, OAra‐cTG	Ranging from 33% to 75% of SD	See above	CR in 1 of 2 on SD, CR in 1 on RD	See above
		Bienemann et al	0	1	III, NB‐RG	NHL‐BFM 86	50% SD in protocol I, protocol M stopped because of severe toxicity, protocol II omitted	Toxicity experienced, not elaborated		
	DLBCL	Sandlund et al	0	5	III, IV	LMB‐89	Group C patients treated according to Group B arm (max MTX dose was 3 g/m^2^)	2 sepsis, 1 pneumonitis, 2 multi‐organ failure, 1 severe VCR peripheral sensory neuropathy, 1 severe pulmonary leak with Ara‐C	2 achieved CR 1 induction failure	
		Yamada et al	0	1		9104 Standard risk protocol by Tokai Pediatric Oncology Study Group	50% of SD	Nil side effects apart from mild reversible liver damage	Remained in CR 32 mo after diagnosis	
		Rossi et al	0	1	IV‐B	Modified dose of R‐CHOP	100% rituximab and prednisolone, 40% CPM, 30% DOX, 70% VCR		Remained in CR 24 mo after diagnosis	
		Bienemann et al	0	8	II‐III	B‐NHL BFM 90, 95, 04	Mostly 50% SD, some gradually uptitrated to 75% and 100% Some limit MTX dose to 0.5‐1 g/m^2^ VP16, IFO, VCR & CPM omitted in some cases	1 died from treatment‐associated toxicity at the end of the fourth course	At least 4 achieved CCR	
		Upadhyaya et al	0	1	IIIB	Modified LMB protocol	Induction phase—50% SD for CPM, DOX, IV MTX, VBL instead of VCR	Fungemia, transaminitis; MTX and DOX further dose reduced due to neutropenia and mucositis		
	T cell	Overberg‐Schmidt et al	1	0		Acute lymphoblastic leukemia‐Berlin, Frankfurt, Munster 86 protocol		Hepatotoxicity, diarrhea, and recurrent varicella and herpes simplex infection; chemo stopped after 5 mo due to life‐threatening complications	Achieved CR	EFS ~ 3 y OS 3 ye 8 mo
	NOS	Sandoval & Swift	1	0	IV	CHOPB		See above	Achieved CR	See above
Hodgkin disease (HD)	Nodular sclerosis (NS)	Tamminga et al	0	1	IIA	Reduced dose OPPA + involved field RT	Procarbazine omitted 1st course 1/3 of SD 2nd course 2/3 of SD 3rd course full dose	Tolerated 1st and 2nd course well NCI grade 3 BM suppression and NCI grades 2‐3 paralytic ileus with 3rd course	Achieved CR	EFS: 3 mo (biopsy non‐conclusive) OS: 10 mo, due to generalized progressive lymphadenopathy and pneumonia
		Upadhyaya et al	0	1	IVB	VAMP/COP Salvage ICE (MD)	VBL instead of VCR, reduced dose of CPM, DOX, MTX	Prolonged myelosuppression, suspected splenic fungal lesions; worsening ataxia with ICE	Achieved CR	2.5 y—due to relapse
	2 NS, 2 lymphocyte depleted	Sandoval & Swift	0	4	IIIB, IVB	HOP/ChVPPr, P + VP, ABVD	Ranging from 33% to 75% of SD	See above	All 4 patients did not achieve remission	See above
	1 NS, 1 NOS	Ben Arush et al	0	2		COPP/ABV	75% of SD VBL instead of VCR was given after the 1st cycle for 1 patient due to toxicity	Severe SIADH and convulsions after first cycle—either due to VCR or CPM	Both achieved CR	
	Mixed cellularity	Irsfeld et al	0	2	IIA, IVB	German Group of Pediatric Oncology—HD 1990 for 1 patient, HD 1995 for 1 patient	1 pt received 3 courses of OPPA instead of 2 in place of radiotherapy 1 pt had ABVD instead of COPP to avoid use of CPM	1 had neurological deterioration? related to VCR 1 had CMV pneumonia presumably due to underlying immunodeficiency	1 achieved CR	
	Classical HD	Bienemann et al	0	1	IVB	Only prednisolone	Only prednisolone			1 mo
Acute Lymphoid Leukemia (ALL)	T‐PLL	Geling Li et al	1			Alemtuzumab 30 mg 3x/wk, pentostatin 4 mg/m^2^, tofacitinib	Tofacitinib dose adjusted according to renal function	Pentostatin stopped after acute renal failure requiring hemodialysis despite aggressive hydration, switched to tofacitinib		~4.5 mo
	ALL	Sandoval & Swift	4	1		CHOPB, POD, POLasp, PODLasp, PO + 6‐MP, P	Ranging from 33% to 75% of SD	See above	CR in 4 of 4 on SD, 1 on RD did not achieve remission	See above
		Toledano & Lange	20	0		A variety of regimes, mainly with VCR, L‐asp, MTX, 6‐MP, DNM, prednisolone/prednisone	NA	2 developed severe infections, 1 had neurological deterioration ?related to VCR		
	18 T‐ALL, 2 B‐cell precursor ALL	Schoenaker et al	11	9 (both B‐cell ALL received MD)		A variety of regime: POG 9404 + CNS RT CCG 1882, regime C + CNS RT ALL IC‐BFM 2002 CCG 5911 Reg.1 ALL‐BFM MR DEXA ALL‐BFM MR PRED ALL‐BFM HR PRED NOPHP ALL 2000 BFM INS 93 BFM ALL 2000 DCOG‐ALL9 Vin, pred, dox, asp	Not standardized Those mentioned include omission of alkylating agents, reduction of MTX dose	4 of 11 on upfront SD had severe toxicity (infections, neuropathy, hemorrhagic cystitis, leukopenia) 2 on upfront MD had sepsis Both B‐cell precursor ALL had toxicities despite upfront MD—1 persistent leukopenia, 1 sepsis	No sig difference in CR rates between upfront SD and MD in T‐ALL: CR in 10 of 11 with upfront SD CR in 7 of 7 with upfront MD	73% vs 57% on upfront SD vs upfront MD
	T‐ALL	Ussowicz et al	0	1		ALL IC‐BFM 2002 protocol For high‐risk chemo then allo‐SCT in view of poor prednisone response on D8 of induction therapy	SD for induction (protocol I) SD for dexamethasone, VCR, L‐asp, DNR 50% dose of CPM and IFO 20% dose of MTX 75% dose of cytarabine Omit Vepesid Modified conditioning chemo pre–allo‐SCT	Toxicities after SCT: Grade IV leucopenia with agranulocytosis, grade II mucositis, multiple viral infections, BKV hemorrhagic cystitis, EBV lymphoproliferative disorder	Remained in complete hematological remission 3.5 y after SCT	
		Bienemann et al	4	2		ALL‐BFM MR DEXA ALL‐BFM MR PRED ALL‐BFM HR PRED	50%‐75% dose for DNR, CPM, DOX, VCR, ASP, ARA‐C, MP Dose reduction up to 20% SD for MTX	Both on MD died of treatment‐associated toxicities	At least 3 of 4 on SD achieved CCR	
	Pre‐B ALL	Brummel et al	0	1		Modified intermediate‐risk group ALL‐BFM‐2000 study protocol	Start with 50% SD Increase to 66% SD for DNR Increase to 75% SD for Ara‐C Increase to 100% SD for VCR, L‐asp Limit CPM to 50% SD, DOX to 66% SD, IV MTX to 20% SD Dexamethasone, IT MTX at 100% SD	Persistent neutropenia with IV MTX Developed pneumonia, candida pelliculosa sepsis, generalized seizures and mutism due to parainfectious encephalitis, recurrent bronchitis	CR on day 15 of therapy Remained in CR > 1 y after end of maintenance therapy	
		Bienemann et al	0	1		ALL‐BFM MR DEXA	50%‐66% SD for VCR, DNR, ASP, CPM, Ara‐C, MP, DOX, CPM; 20% SD for MTX		Remained in CCR 3.5 y after diagnosis	
Acute Myeloid Leukemia (AML)		Schoenaker et al	1	2		POG‐AML97A prot (SD), ECM‐HCEI course (MD), Oral 6‐MP (palliative)		Both SD and palliative patients died of sepsis	SD—did not achieve remission MD—achieved CR	
		Onoda et al	0	1			Low‐dose induction therapy ‐ VP16 100 mg/m^2^ D1‐3, Ara‐C 150 mg/m^2^ D1‐3, DNR 25 mg/m^2^ D1, IT MTX 15 mg/dose D1 Dose optimized due to increased AML blasts ‐ 2 courses induction + 3 courses intensification based on high dose cytarabine (HiDAC) with CNS prophylaxis	Alpha‐hemolytic streptococcal sepsis and pneumonia during second induction, transiently requiring non‐invasive positive pressure ventilation, irreversible unilateral pleural effusion	Hematological remission after induction phase	~1 y, due to respiratory failure and leukemia relapse
Solid tumors	Nephroblastoma	Perez‐Villena et al	0	1	III	SIOP‐TW‐01 protocol	25% dose reduction, omitted radiotherapy	Staphylococcus epidermidis bacteremia Septic shock after topotecan given for relapse	Relapse at 34th week of treatment (during last cycle)	40 wk
	Dysgerminoma	Koksal et al	0	1		Carboplatin 450 mg/m^2^ D1, VP16 100 mg/m^2^ D1‐3, bleomycin 10 mg/m^2^ D2	Regime as stated instead of first‐line PEB to avoid use of cisplatin	Developed pneumonia after 1st cycle Lung function deterioration—bleomycin stopped after 1 cycle	No evidence of residual or recurrence mass at second year of diagnosis	
		deVries & Kaplan	1	0	IIIc	Cisplatin + vinblastine x 2 cycles			No sign of recurrence at 24 mo postdiagnosis	
	Endodermal sinus of the ovary	Pecorelli et al	0	1	Ic	Cis‐platinum, vinblastine, bleomycin x 5 courses postoperatively	50% of SD	WHO grade 2 neurotoxicity at the 4th course of treatment	Remained in remission 20 mo after treatment	

Abbreviations: 6‐MP, 6‐mercaptopurine; ABVD, doxorubicin, bleomycin, vinblastine, dacarbazine; Ara‐C, cytarabine; CCR, complete clinical remission; CHOP, cyclophosphamide, doxorubicin, vincristine, prednisone; CHOPB, cyclophosphamide, doxorubicin, vincristine, prednisone, bleomycin; CHVP, cyclophosphamide, doxorubicin, etoposide; CO, cyclophosphamide, vincristine; COP, cyclophosphamide, vincristine, prednisone; COVM, doxorubicin, vincristine, vinblastine, methotrexate; CPM, cyclophosphamide; CR, complete remission; DNM, daunomycin; DNR, daunorubicin; DOX, doxorubicin; EFS, event‐free survival; HOP, doxorubicin, vincristine, prednisone; HOP/ChVPPr, doxorubicin, vincristine, prednisone/chlorambucil, vinblastine, prednisone, procarbazine; IFO, ifosfamide; L‐asp, L‐asparaginase; M, methotrexate; MD, modified dose; MP, Mercaptopurine; MTX, methotrexate; NOS, not otherwise specified; OAra‐cTG, vincristine, cytarabine, thioguanine; OH, vincristine, doxorubicin; OP, vincristine, prednisone; OPPA, vincristine, prednisone, procarbazine, doxorubicin; P, prednisone; PEB, bleomycin, etoposide, cisplatin; PODLasp, prednisone, vincristine, daunomycin, asparaginase; RD, reduced dose; SD, standard dose; VBL, vinblastine; VCR, vincristine; VP, P, etoposide, prednisone; VP16, etoposide.

^a^ Based on all patients in the study (regardless of tumor types).

Given significant considerations in the management of malignancies, early diagnosis of A‐T, prior to that of malignancy should there be, is of critical importance. Although there are no guidelines for cancer screening in A‐T, early diagnosis and hence knowledge of the underlying genetic disorder will allow patients/families to be cognizant of symptoms and prompt clinicians to do the necessary screening and workup, hopefully enabling detection of malignancy, if any, at a more favorable stage. Additionally, allogeneic hematopoietic stem cell transplantation has been shown to correct immunodeficiency and potentially retard deterioration of neurological function in case reports^50^, ^51^ which may be considered in selected cases. Regardless, early diagnosis of A‐T also allows for earlier introduction to a multidisciplinary care team,^7^, ^52^ with the aim to reduce associated morbidities, such as reducing contractures and maintaining functional activity, improving airway clearance, reducing aspiration risk, appropriate treatment of infections especially if recurrent, earlier detection and management of endocrinopathies, ultimately improving quality of life.

## 4 CONCLUSION

There is a need to improve the general genetic literacy of all clinicians. Ataxia‐telangiectasia is one of the important hereditary cancer syndromes that clinicians should not only be aware of, but also be astute in identifying the diagnostic clues. Any co‐occurrence of neurodevelopmental diagnosis must trigger a consideration for timely genetic testing. Also, AFP should be measured to rule out A‐T in children and patients with progressive neurological decline. Early diagnosis is critical as it may significantly alter management, treatment approach in individuals diagnosed with cancer and allow for interventions that may potentially reduce associated morbidities.

## CONFLICT OF INTEREST

We have no conflict of interest to disclose.

## AUTHOR CONTRIBUTION

JC: wrote the manuscript. RT: revised the manuscript and contributed to the interpretation of data. STL: revised the manuscript and contributed to the interpretation of genetic data. EC: revised the manuscript and contributed to the interpretation of genetic data. RG: revised the manuscript and contributed to the interpretation of pathological data. EF: revised the manuscript and contributed to the interpretation of data. KS: revised the manuscript. RN: revised the manuscript. JN: contributed to the interpretation of data, revised, and oversaw the writing of the manuscript.

## INFORMED CONSENT

Our patient verbally consented to the publication of this case report. Written consent was provided by patient's mother on behalf of patient due to physical disability.

## EDITORIAL POLICIES AND ETHICAL CONSIDERATIONS

Approval by our Centralised Institutional Review Board is not required for case report.

## Data Availability

Data sharing is not applicable to this article as no new data were created or analyzed in this study.

## Appendix 1 Cancer spectrum of A‐T individuals

**Table:**

Majority of malignancies in childhood are leukemias and lymphomas T‐cell tumors > B‐cell tumors (unlike majority of childhood leukemias in non‐A‐T patients which are pre‐B‐cell leukemias) Myeloid tumors are rare Young adult A‐T patients predisposed to T‐PLL Adult A‐T patients are still at risk of lymphoid tumors but also has increased incidence of solid tumors

## Appendix 2. Chemotherapeutic agents of concern in A‐T and recommendations

**Table:**

Chemotherapeutic agents	Concerns	Suggested approaches from studies
Alkylating agents	Acts by inducing DNA breaks	Avoid use or consider dose reduction
Bleomycin	Increased risk of pulmonary toxicity despite being administered at a reduced dose	Avoid use
Cyclophosphamide/ifosfamide	Higher risk of hemorrhagic cystitis, thought to be related to the presence of telangiectasia in the bladder	Limit dose to <1200 mg/m^2^, ensure vigorous hydration and concurrent mesna administration
Methotrexate	Neutropenia and infections. Gastrointestinal tract toxicities in children	Consider starting at reduced dose, up titrate as tolerated. Aggressive hydration, appropriate alkalinization of urine to optimize clearance and use of rescue leucovorin. Close monitoring of methotrexate levels
Topoisomerase II inhibitors	Acts by inhibiting repair of DNA double‐stranded breaks	Consider dose reduction
Vinca alkaloids	May worsen or confound progression of underlying neurological status	Consider alternatives, reduced dose and omission in event of neurological deterioration
